# Impact of Flexible Circuit Bonding and System Integration on Energy Resolution of Cross-Strip CZT Detectors

**DOI:** 10.1109/trpms.2023.3256406

**Published:** 2023-03-22

**Authors:** Emily Enlow, Milad Diba, James Clayton, Brian Harris, Shiva Abbaszadeh

**Affiliations:** Department of Electrical and Computer Engineering, University of California Santa Cruz, Santa Cruz, CA 95064 USA; Department of Electrical and Computer Engineering, University of California Santa Cruz, Santa Cruz, CA 95064 USA; Polymer Assembly Technology Inc., Rockford, MI 49341 USA.; Kromek, Zelienople, PA 16063 USA.; Department of Electrical and Computer Engineering, University of California Santa Cruz, Santa Cruz, CA 95064 USA

**Keywords:** Cadmium zinc telluride (CZT), cross-strip electrode configuration, electronics noise, energy resolution, gold stud bonding

## Abstract

Cadmium zinc telluride (CZT) detectors enable high spatial resolution and high detection efficiency and are utilized for many gamma-ray and X-ray spectroscopy applications. In this article, we describe a stable bonding process and report on the characterization of cross-strip CZT detectors before and after bonding to flexible circuit. The bonding process utilizes gold stud bonding and polymer epoxy technique to bond the flexible circuits to two CZT crystals and form a detector module in an anode-cathode-cathode-anode (ACCA) configuration. The readout electronics is optimized in terms of shaper setting and steering electrode voltage. The average full-width half maximum (FWHM) energy resolution at 662 keV of 110 CZT crystals tested individually was 3.5% ± 0.59% and 4.75% ± 0.48% prebonded and post-bonded, respectively. No depth correction was performed in this study. The average FWHM energy resolution at 662 keV of the scaled-up system with 80 CZT crystals was 4.40% ± 0.53%, indicating the scaled-up readout electronics and stacking of the modules does not deteriorate performance. The proper shielding and grounding of the scaled-up system slightly improved the system-wide performance. The FWHM energy resolution at 511 keV of the scaled-up system was 5.85% ± 0.73%.

## I. Introduction

CADMIUM zinc telluride (CZT) is a compound semiconductor material with room temperature operation which has been utilized in different fields, including nuclear medicine, X-ray and gamma-ray astronomy, and homeland security. In comparison to the scintillators and gas detectors, CZT detectors present superior energy resolution and high spatial resolution with simple pixel electrode configuration [1], [2], [3]. In addition, the cost of CZT detectors per cm^3^ has been reduced over the past 15 years, leading to its rapid adoption by multinational original equipment manufacturers (OEMs), for example single-photon computed tomography (SPECT) cameras introduced by GE Healthcare [4].

Pixel-array and cross-strip electrode configurations are two common electrode designs for CZT crystals. Compared to cross-strip, pixellated configuration requires a high number of readout channels to cover the same area (*N*^2^ versus 2*N*). High density channels increase the cost of the readout electronics especially when it comes to integration of large-scale electronics for applications, such as PET and SPECT. In cross-strip detector configuration, anodes and cathodes are perpendicular to each other which offers a high packing fraction (99%) by enabling tight stack of crystals on top of each other and lower number of readout channels [5], [6], [7], [8]. In some applications, the reduced complexity of the readout electronics, low cost, and high packing fraction make cross-strip configurations advantageous at the cost of higher capacitance and lower energy resolution compared to the pixel-array design [9], [10].

In this work, we investigate the effects of flexible circuits used for bonding the cross-strip CZT crystals to the readout electronics by evaluating the energy resolution prebonding, post-bonding, and in a scale-up configuration.

## II. Materials and Methods

**Fig. 1.**
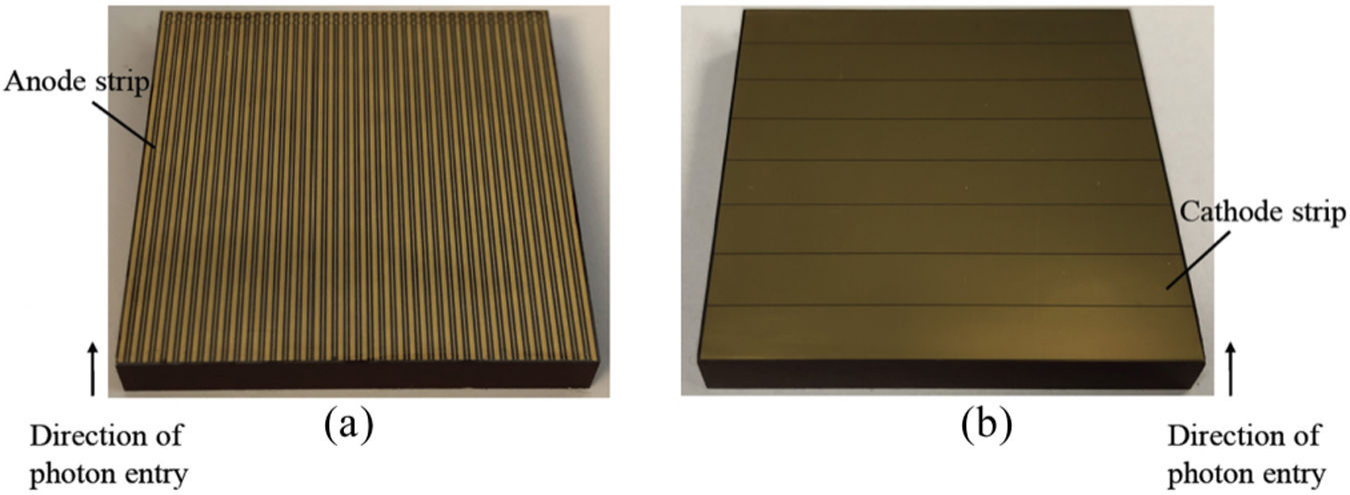
(a) Anode with steering electrodes, and (b) Cathode layouts of the CZT cross-strip detectors. The anode strips (1-mm pitch; 100-*μ*m width) are orthogonal to the cathode strips (5-mm pitch; 4900-*μ*m width). The steering electrode is 400-*μ*m width.

The cross-strip configuration utilized in this work consists of 39 anode strips (100-*μ*m width and 1-mm pitch), 8 cathode strips (4900-*μ*m width and 5-mm pitch) and 38 steering electrode strips (400-*μ*m width). The use of the steering electrode aims to enhance anode cross-strip charge collection and to improve energy resolution. Fig. 1 represents a CZT crystal with cross-strip anode and cathode electrode pattern. To develop a compact design where CZT crystals can be stacked on top of each other, two CZT crystals build one CZT module in an anode-cathode-cathode-anode (ACCA) configuration. Each Crystal is bonded to flexible circuit and gold stud bond and polymer epoxy technique is used as the electrical connection material between the CZT metal electrodes and flexible circuits. The bonding process of a dual-stack CZT detector module to flexible circuits is discussed in Section II-B.

The ACCA configuration simplifies the alignment process for attaching two crystals together since cathodes (5-mm pitch; 4900-*μ*m width) are wider compared to anodes (1-mm pitch; 100-*μ*m width). The flexible circuit attached to CZT crystals are connected to the board that hold RENA application specific integrated circuit (ASIC). RENA ASIC [11], [12] is a low-noise, 36-channel, self-trigger, self-resetting charge-sensitive amplifier/shaper ASIC developed by NOVA Research and Development (Riverside, CA).

**Fig. 2.**
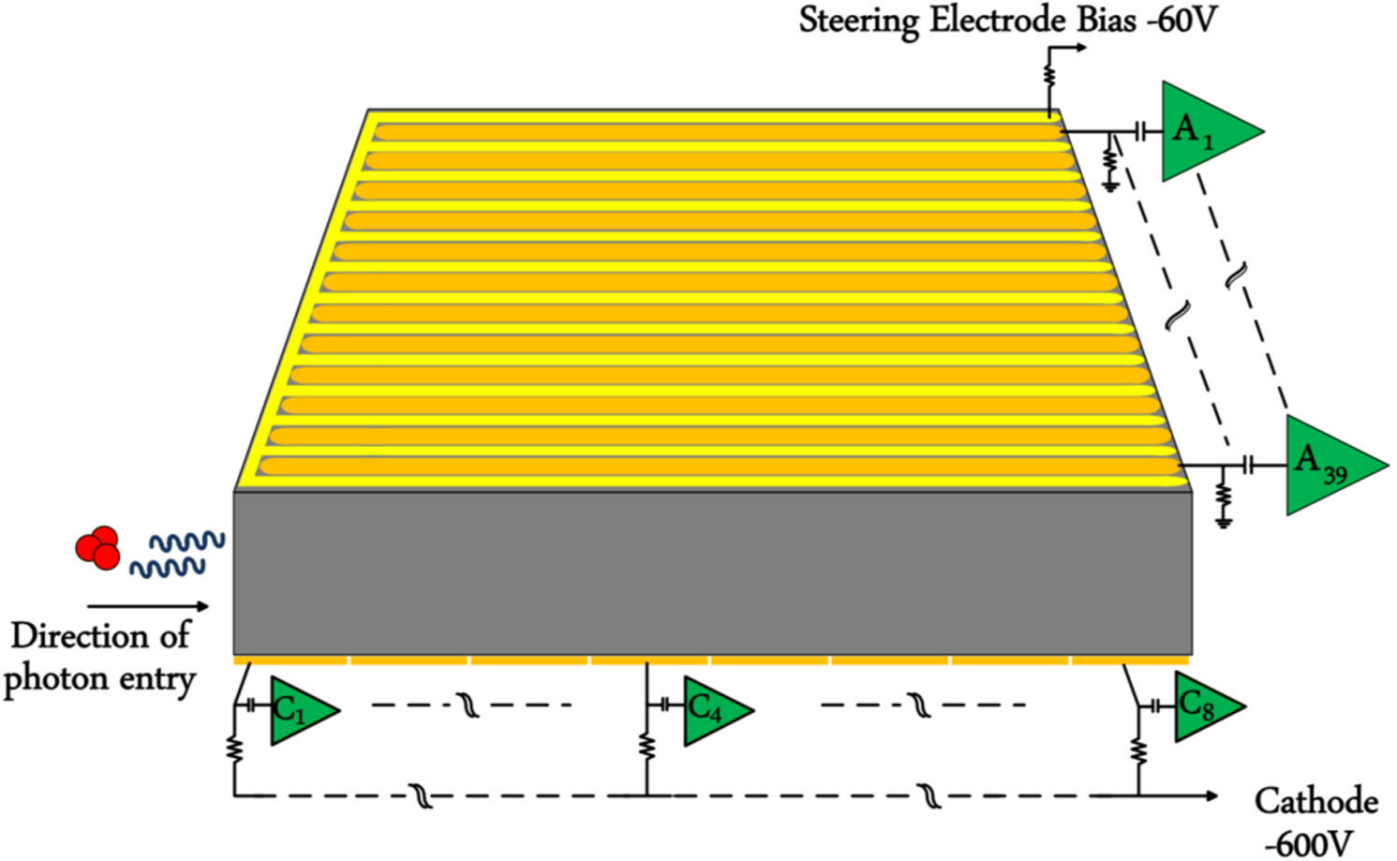
Illustration of Readout front-end. Anode and cathode strips are connected to intermediate board for biasing voltage and RENA board for signal readout. Detailed descriptions of intermediate board and RENA board can be found in [6] and [14].

Fig. 2 summarizes the readout interface to the cross-strip CZT crystal used in this work. The RENA is capable of reading both anode and cathode signals. For this application, RENAs are operated in fast-triggering mode and output of peak-detect and hold (PDH) block is followed by a 12-bit analog-to-digital converter (ADC) and an FPGA to establish communication with computer. For a CZT crystal with more than 36 channels, more than one RENA ASIC will be used. In the current design, each CZT crystal with 39 anodes and eight cathodes utilizes two RENA ASICs. A more detailed description of a modular readout design of cross-strip CZT detectors utilizing RENA ASICs can be found in [13] and [14].

### A. Design of Flexible Circuit

**Fig. 3.**
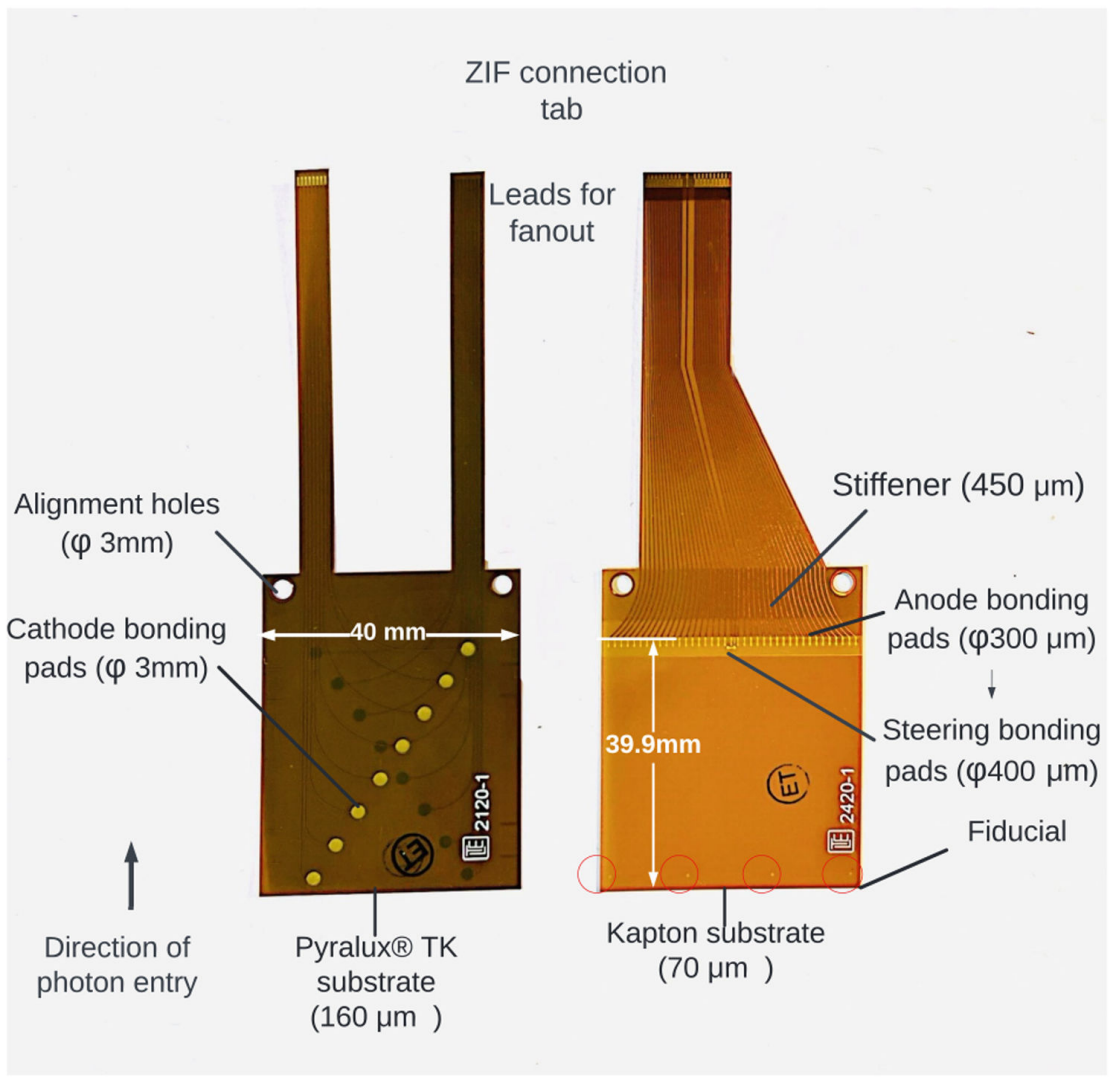
Flexible circuit dimensions for anode (right) and cathode (left).

**Fig. 4.**
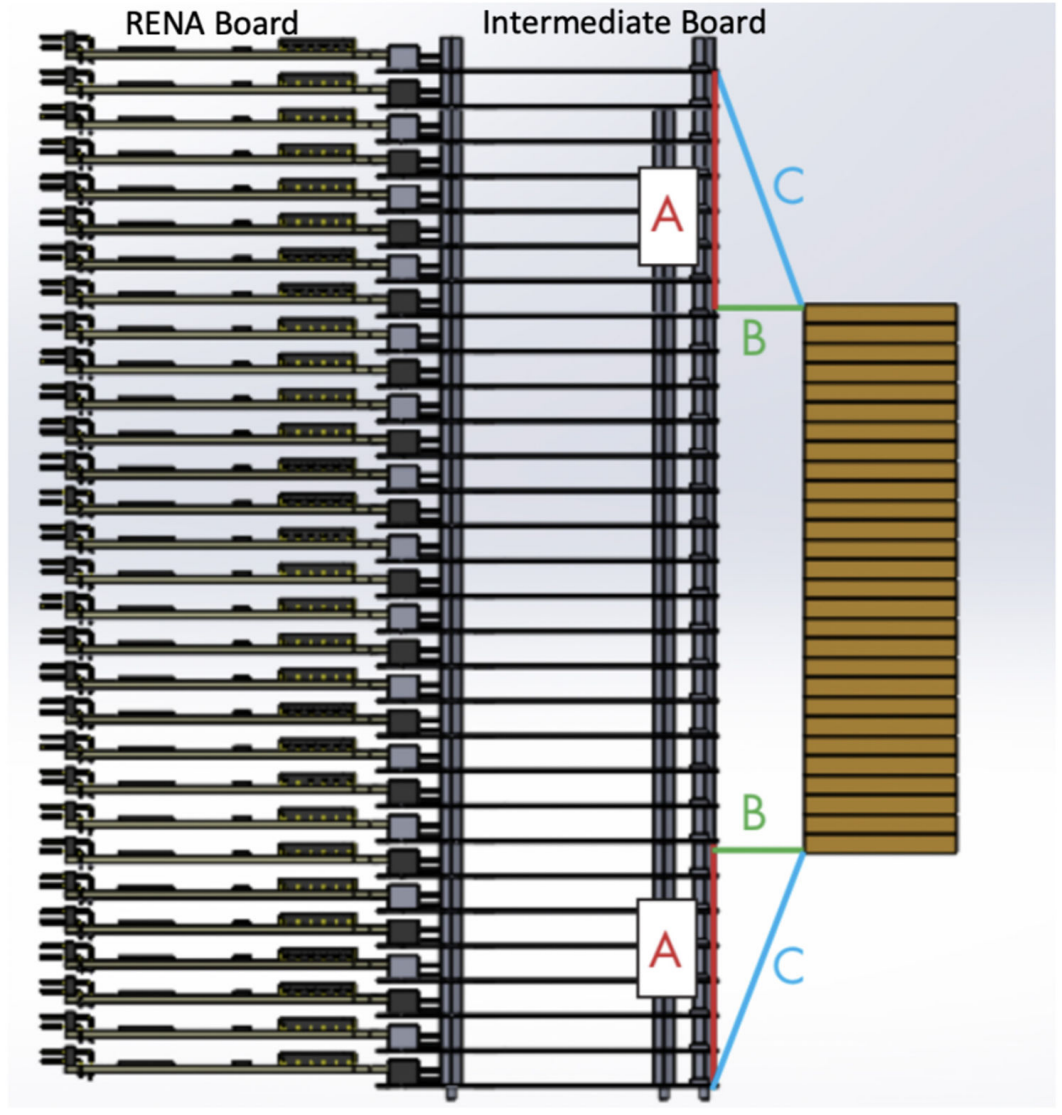
Illustration of the stack of readout boards and CZT crystals indicating larger height of readout boards compared to CZT stack. A is the maximum difference in height between the two stacks of CZT crystals and readout electronic, B is the selected distance of crystals to readout board, and C is the minimum length of flexible circuit.

The anode flexible circuit includes anode bonding pads similar to the anode electrode pattern on CZT crystals (Fig. 3). The minimum length of the flexible circuit is designed based on the number of CZT crystals that is required to be stack on top of each other. Fig. 4 illustrates the minimum length of flexible circuit (C) based on the selected distance of crystals to readout board (B) and half the difference in height between the two stacks of CZT crystals and readout electronics (A). The height of 30 CZT crystals (with 0.5-cm thickness) is 15 cm. The height of the readout board that will be connected to one CZT crystal via flexible circuit is 0.9 cm due to thickness of the printed circuit board, sockets, and RENA ASIC that are assembled on the board. As a consequence, the recommended length of flexible circuit was chosen 5.8 cm assuming parameter B to be approximately 1 cm. The capacitive coupling between overlapping cathodes due to the ACCA configuration will be higher than compared to overlapping anodes in a cathode-anode-anode-cathode configuration. However, ACCA configuration allows simpler inspection and visual access, and troubleshooting. In order to minimize the effect of capacitance coupling, the flexible circuit is a polyimide composite known as DuPont Pyralux [15] that has a smaller permittivity (2.3 to 2.6) compared to Kapton (2.8). In addition to further reduce the capacitance, the thickness of cathode flexible circuit (around 254 *μ*m) is higher than anode flexible circuit (64 *μ*m). The final capacitance between overlapping cathode strips is approximately 15 pF.

### B. Bonding Flexible Circuit to Dual-Stack CZT Crystals

**Fig. 5.**
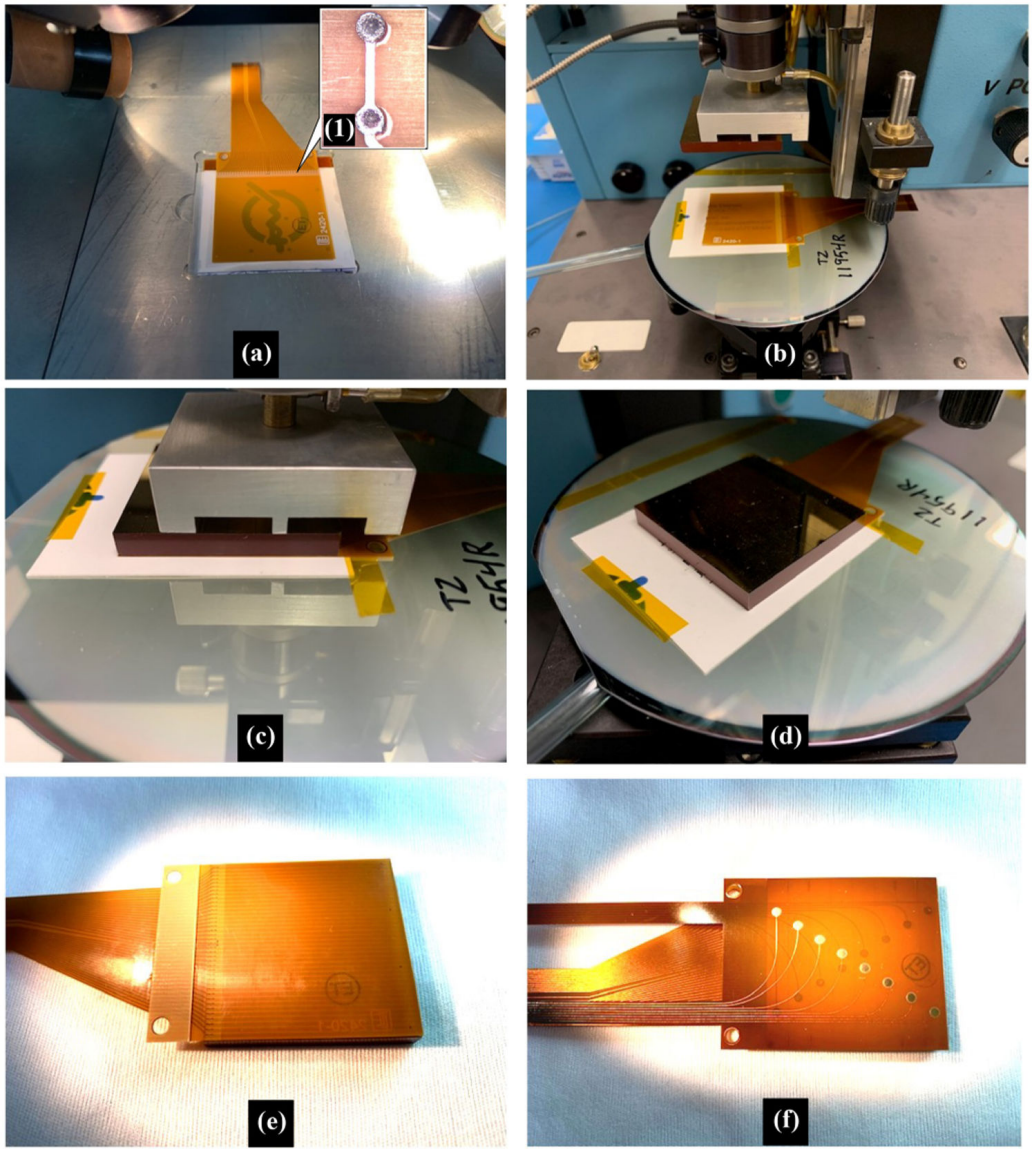
Bonding process. (a) Anode flexible circuit prepared for stencil printing of the epoxy bumps, with magnified view of the silver epoxy bumps on the interconnect pads. (b) Anode flexible circuit is mounted onto a wafer and placed onto the vacuum stage of a flip-chip bonder. The CZT crystal is held by a vacuum fixture with the anode pads facing down and are then aligned to the pads of flex circuit using a split prism. (c) After alignment, the CZT is lowered onto the flexible circuit and released from the vacuum fixture. (d) Wafer is then released from the vacuum stage. (e) Second CZT crystal is assembled in similar manner to an anode flexible circuit. (f) One CZT crystal is then turned over with cathode surface faced up. Epoxy bumps are dispensed onto the bottom pads of the cathode flex circuit before aligning it and placing it onto the CZT crystal. More epoxy bumps are then dispensed onto the pads of the upper surface of the cathode flex circuit.

Gold stud bonding with polymer epoxy, metal bump bonding techniques, and anisotropic conductive film (ACF) have been utilized for bonding of pixellated CZT crystals and readout board [16], [17]. Pixel configuration, size of the boding pad and their pitch, electrode metallization, and operative voltage are important factors to consider for bonding. A comparison study of the common type of ACF used demonstrated higher dark current performance. As a consequence, we have chosen Gold stud bonding with polymer epoxy for bonding CZT crystals to flexible circuit. Electrically conductive epoxy bumps are stencil printed directly onto the total bond pads on the anode side of the CZT detectors before flip chip alignment and placement onto the anode flexible circuits. The mass of the CZT detector aids in compressing the epoxy bumps against the bonding pads on the anode flexible circuit and keeping the flexible circuit flat during the curing cycle of the epoxy bumps. A low-temperature cure (70 °C) also aids in minimizing misalignment due to coefficient of thermal expansion (CTE) mismatch of the materials being bonded together. After curing, the alignment of the interconnections of the epoxy bumps with the pads of the anode flexible circuit can be closely examined under a microscope. Fig. 5 shows the described bonding setup and steps.

Next, the anode flexible circuits require two strips of polyimide adhesive tape to be manually applied at both ends of the CZT detector to prevent the flexible circuit from being accidentally lifted and potentially causing the epoxy bonds to be over-stressed. Since the cable portion of the flexible circuits extends beyond the end of CZT detector where the epoxy bonds are located, a single-sided strip of polyimide tape is applied to the right-angle surfaces along the underside of the flexible circuit and side-wall of the detector. At the opposite end of the detector, the flexible circuit has to be carefully raised without overstressing the epoxy bonds to insert a thin strip of double-sided polyimide adhesive tape to adhere the flexible circuit along the full length of the end.

The final assembly of the dual-stacked detectors requires two detectors to be joined with a single cathode flexible circuit sandwiched between the cathode side of both detectors, with the cable portion extending from the same end of the assembly as the anode flexible circuits. The cathode flexible circuit has eight conductive pads and traces on both sides of the circuit for electrical connection to the eight electrode strips on the CZT detectors. This assembly step requires epoxy bumps to be deposited onto the pads of the first side of the cathode flexible circuit before flip chip alignment and placement of the first CZT detector. After curing and microscopic inspection, epoxy bumps are again applied to the second side of the cathode flexible circuit and the second detector is flip chip aligned and placed onto the preassembled first CZT detector. Since the cathode flexible circuit is sandwiched between the two CZT detectors, no microscopic inspection is possible for this final step of the assembly.

### C. Cross-Strip CZT Detector Characterization

**Fig. 6.**
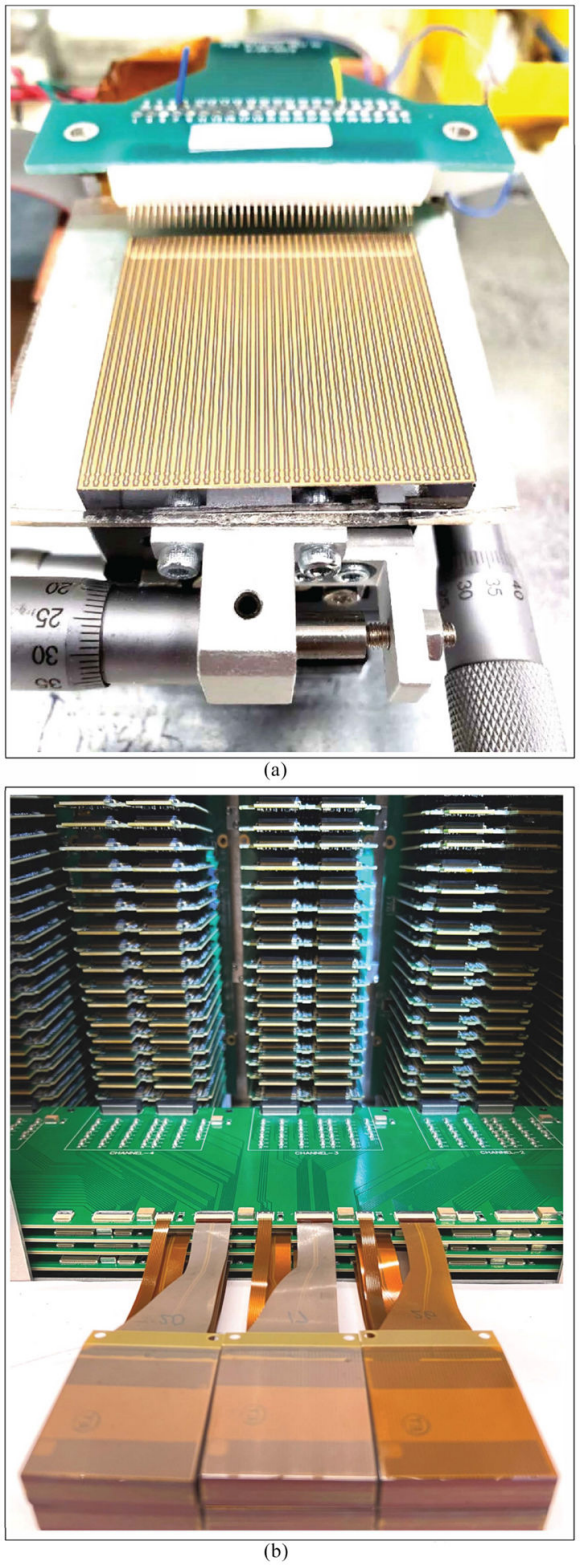
(a) Prebonded CZT measurement set-up with a custom-designed probe card. (b) Post-bonded CZT measurement set-up with RENA board and biasing intermediate board.

In order to evaluate the energy resolution of the detector before and after bonding to the flexible circuits, two experimental setups were utilized (Fig. 6). Fig. 6(a) is the experimental setup used by Kromek. The CZT crystals are directly placed on a printed circuit board and probed by a direct engagement of a custom-designed probe card for measurement at the manufacturer. Fig. 6(b) is the modular design used for the scale-up system [14]. Anodes were biased at 0 V, cathodes were biased at −600 V, and steering electrodes’ bias was optimized and kept at −60 V.

RENA ASIC has 16 shaper setting starting from 0.29 to 38 *μ*s. To determine the optimum setting, we have swept the peaking time starting from 0.79 to 4.5 *μ*s. After optimization, the shaper setting was kept at 1.1 *μ*s.

We conducted measurements using ^137^Cs (662 keV) and ^68^Ge (511 keV) radiation sources. Individually, 110 CZT were tested and the average full-width-half-maximum (FWHM) energy at 662 keV for prebonded and post-bonded crystals were measured. Prebonded energy resolution at 511 keV was not provided by manufacturer. The average FWHM energy resolution at 511 keV for the post-bonded crystals were measured. For the measurements of post-bonded crystals, the activity of ^137^Cs and ^68^Ge was 46.8 and 7.1 *μ*Ci, respectively. The ^137^Cs test duration was 15 min, while the ^68^Ge test duration was 75 min. The channel calibration to obtain photon energies from the RENA ADC values was conducted for all electrodes using 511 and 662 keV for post-bonded experiment.

## III. Results

### A. Steering Electrode Bias

**Fig. 7.**
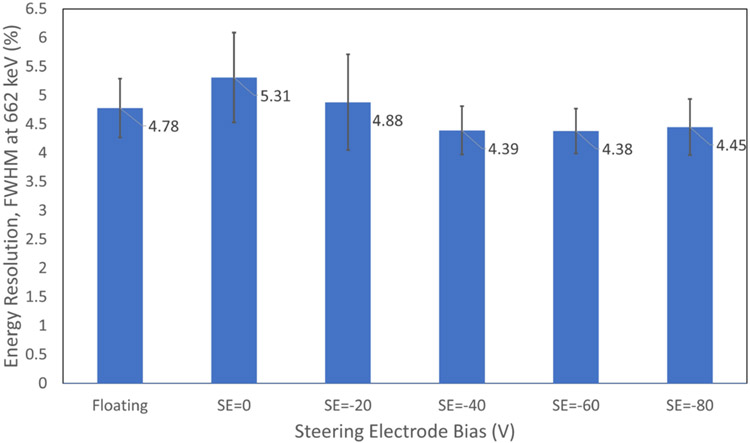
Average FWHM energy resolution as a function of steering electrode bias voltage. Error bars represent standard deviation for average energy resolution measurement.

The steering electrodes are 400-*μ*m width strips spaced between the anode electrodes. Fig. 7 shows the energy resolution performance of the detector with respect to different steering electrode bias voltages while the cathode strips are kept at −600 V and the anode strips are kept at 0 V. The steering electrode bias was varied from floating (no applied bias), 0, −20, −40, −60, and −80 V. Three CZT crystals each with 39 anode channels were tested at 662 keV. The minimum average FWHM energy resolution was 4.38% ± 0.39% when a bias of −60 V was used.

### B. RENA ASIC Shaper Setting

**Fig. 8.**
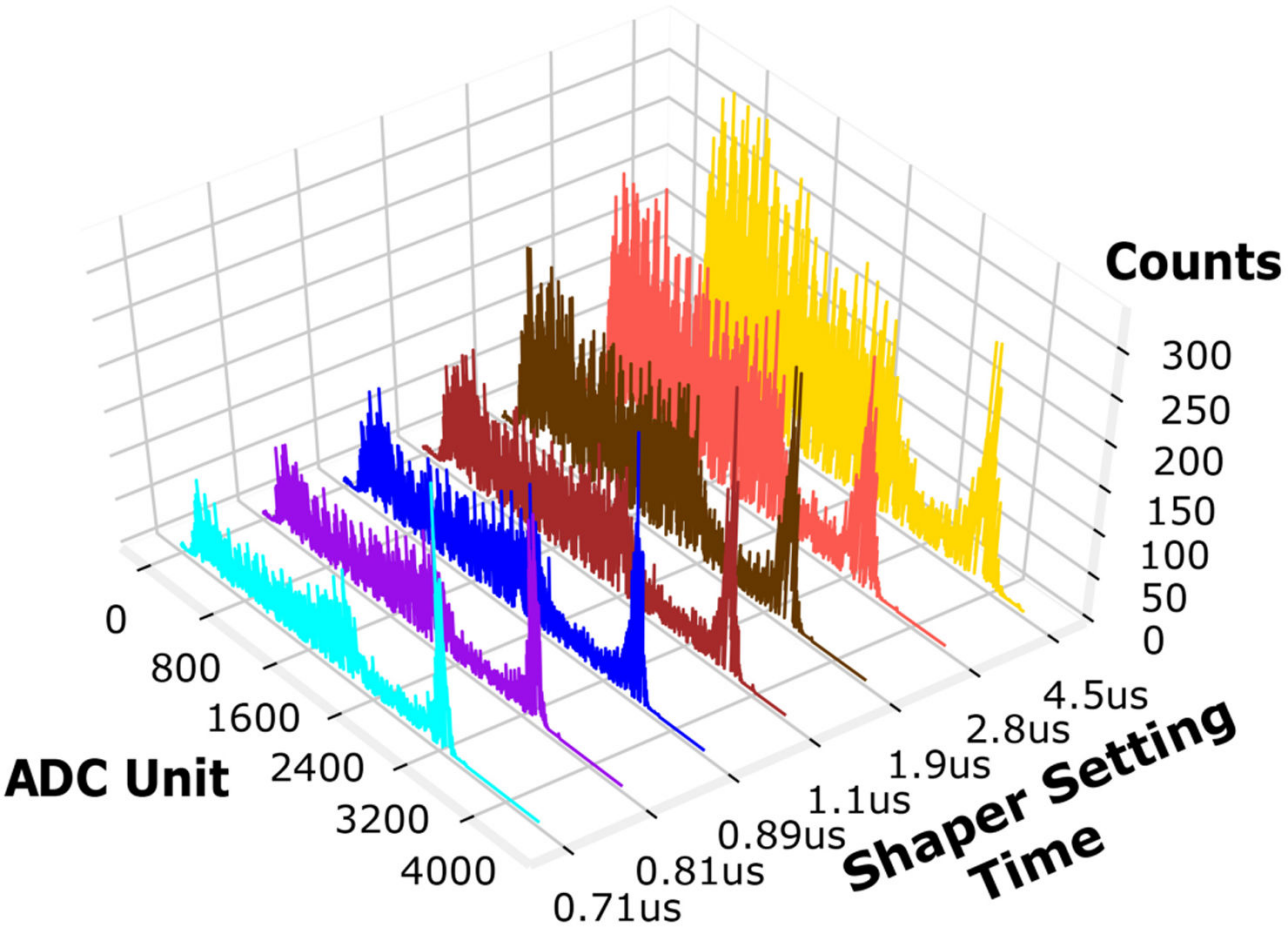
Cs-137 energy spectra versus RENA ASIC shaper setting time.

**Fig. 9.**
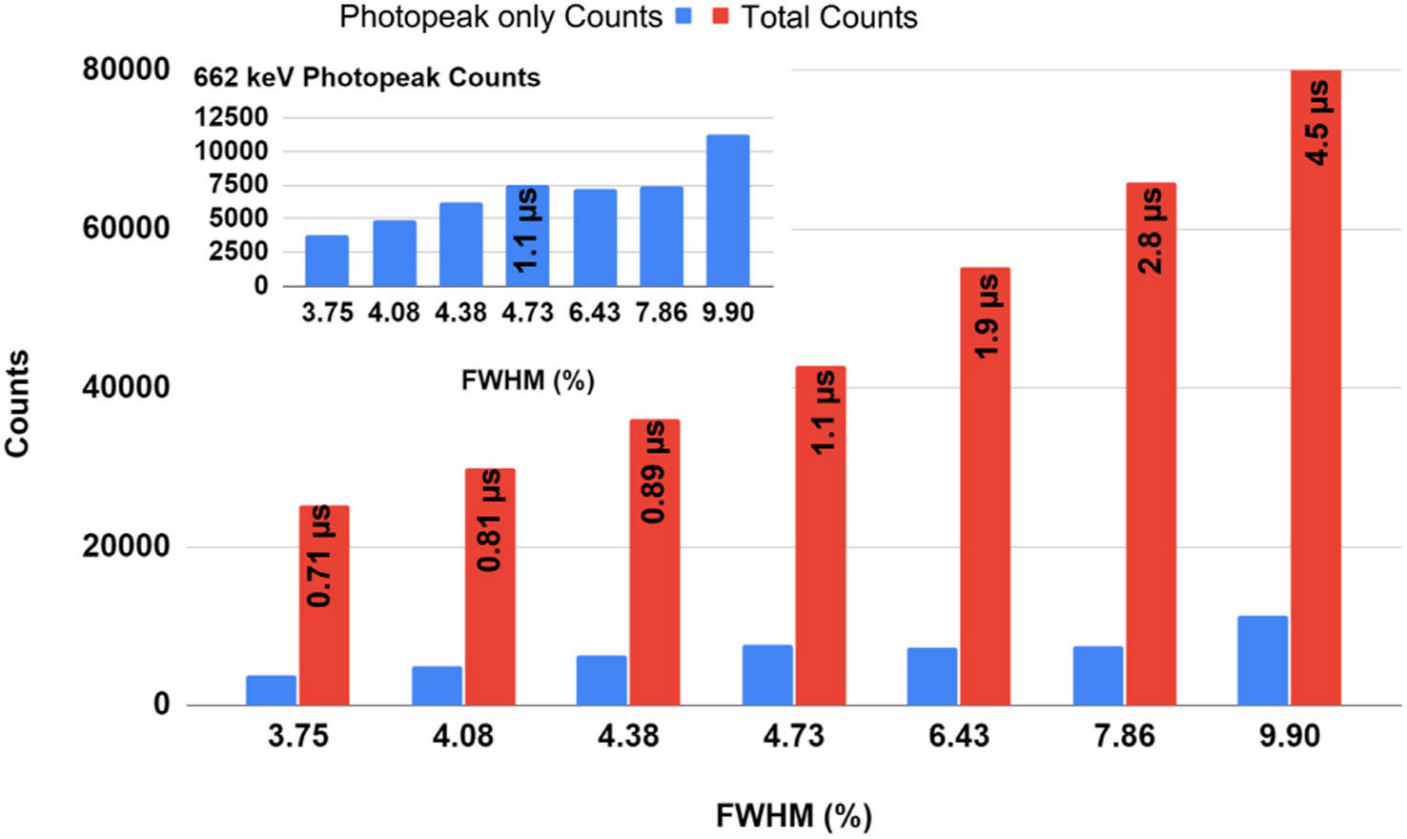
Photopeak counts for 662 keV only (blue), Total counts (red) versus FWHM values for different shaper setting times. Insert: Photopeak counts for 662 keV only, zoomed in.

The time required for the signal to arise from the baseline to its peak value is called the peaking time and is a critical parameter in front-end circuit design [18], [19], since it contributes both to speed and noise of the system. The RENA ASIC is a 36 channel signal processor which contains “slow” and “fast” amplifiers that operate in parallel for energy and timing measurements, respectively. The “slow” shaping amplifier has variable settings that were changed to test the effect of shaper settings on the energy resolution [11]. Fig. 8 shows the ^137^Cs energy spectrum versus RENA shaper setting. Fig. 9 shows the sum of total counts (red) and photopeak-only counts (blue). The shaper setting of 1.1 *μ*s resulted in the largest fraction of photopeak counts to total counts.

### C. Energy Resolution of Individual CZT Crystals

**Fig. 10.**
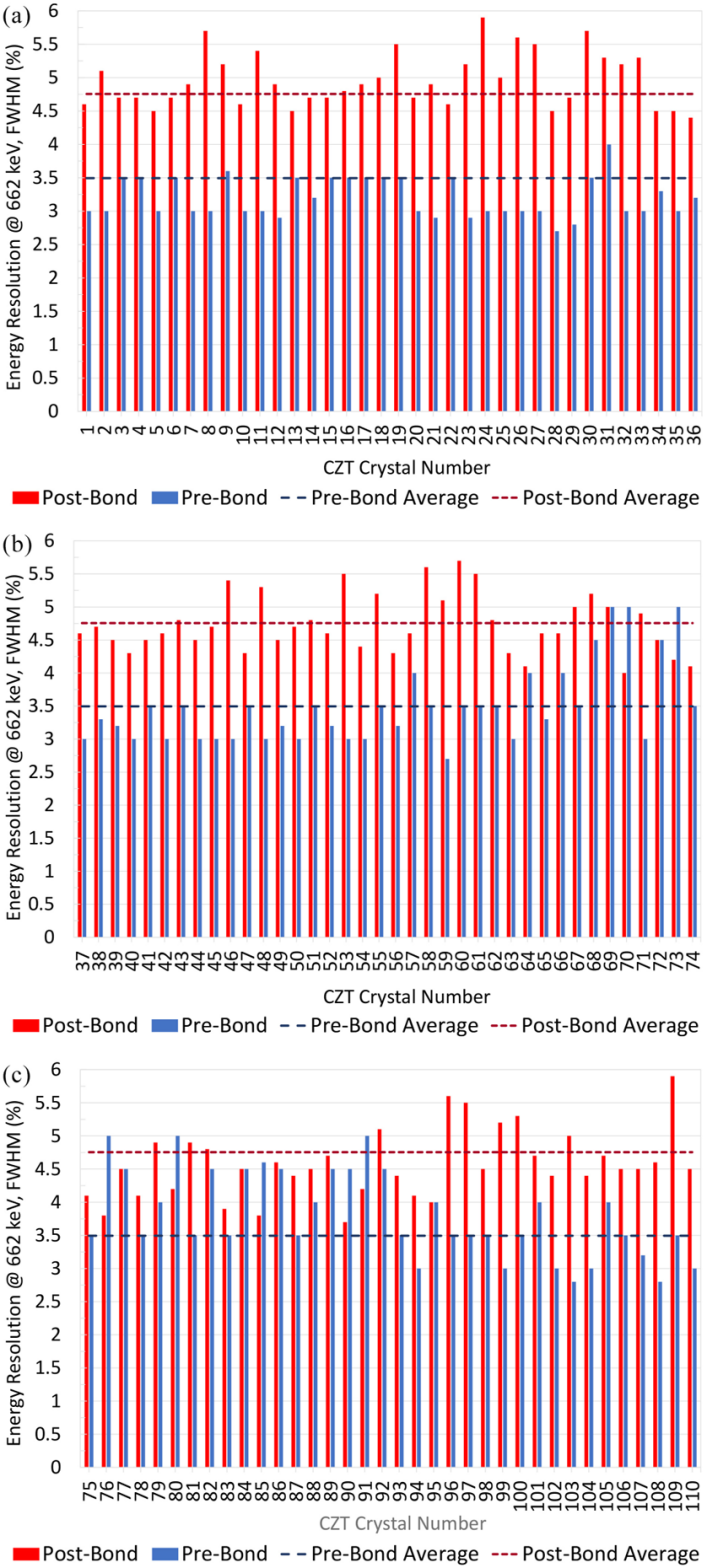
Energy resolution at 662 keV for 110 CZT crystals measured prebond and post-bond process. (a) 1–36, (b) 37–74, and (c) 75–110. Average energy resolution prebond 3.50%, post-bond 4.76%.

Fig. 10 shows a comparison of the energy resolution (at 662 keV) for 110 CZT crystals prebonded measured with a custom-designed probe card and post-bonded. The average energy resolution at 662 keV for prebonded and post-bonded crystals was 3.50% ± 0.59% and 4.76% ± 0.48%, respectively. This shows an increase in energy resolution of 1.26%, which is likely caused by a combination of the flexible circuit bonding and the additional electronics. The post-bonded modules were measured at 511 keV and the average energy resolution was 5.82% ± 0.59%. The manufacturer did not supply energy resolution at 511 keV.

### D. Energy Resolution of Scaled-Up System

**Fig. 11.**
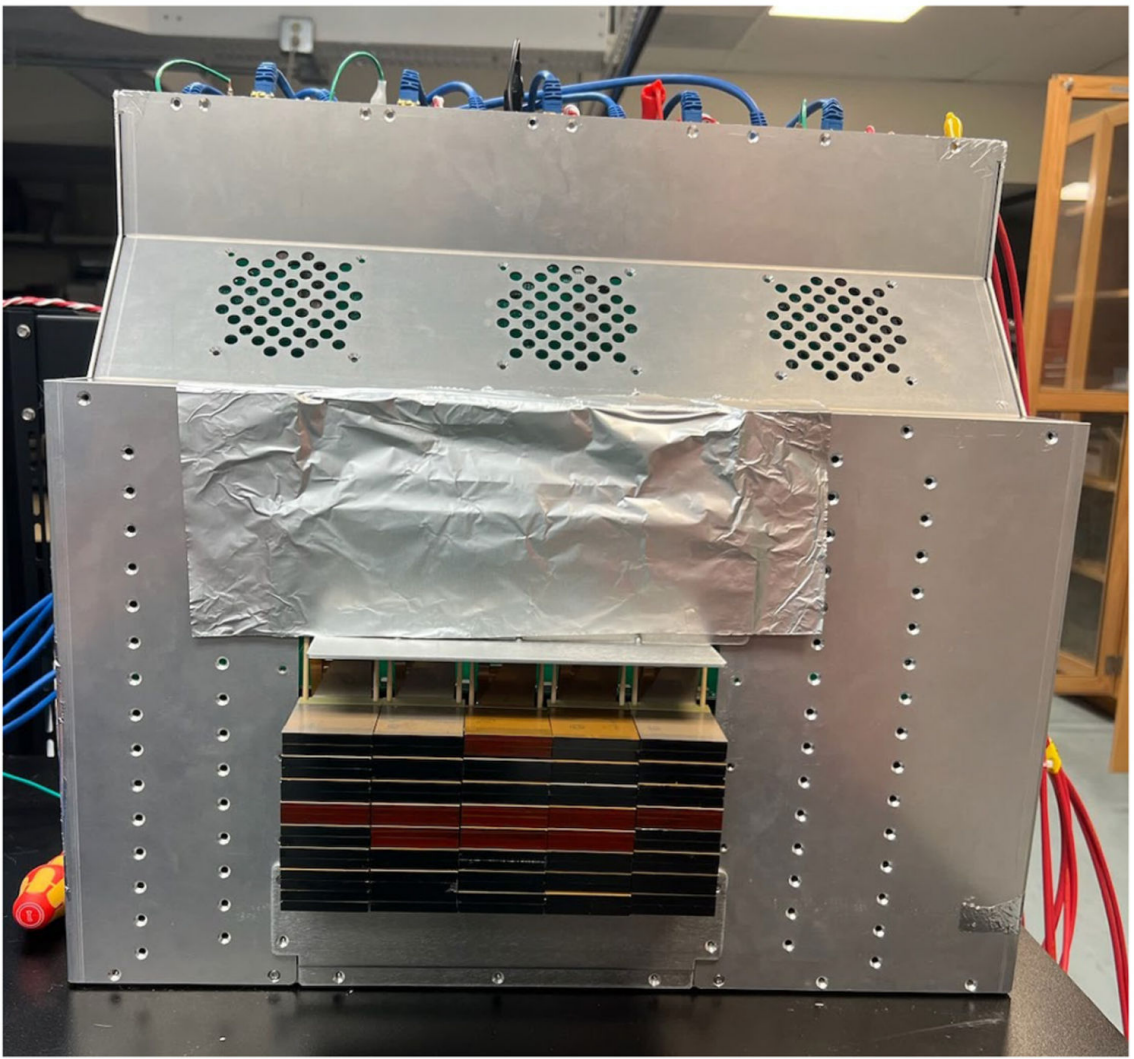
Scaled-up system with 80 CZT crystals installed in Panel 1.

**Fig. 12.**
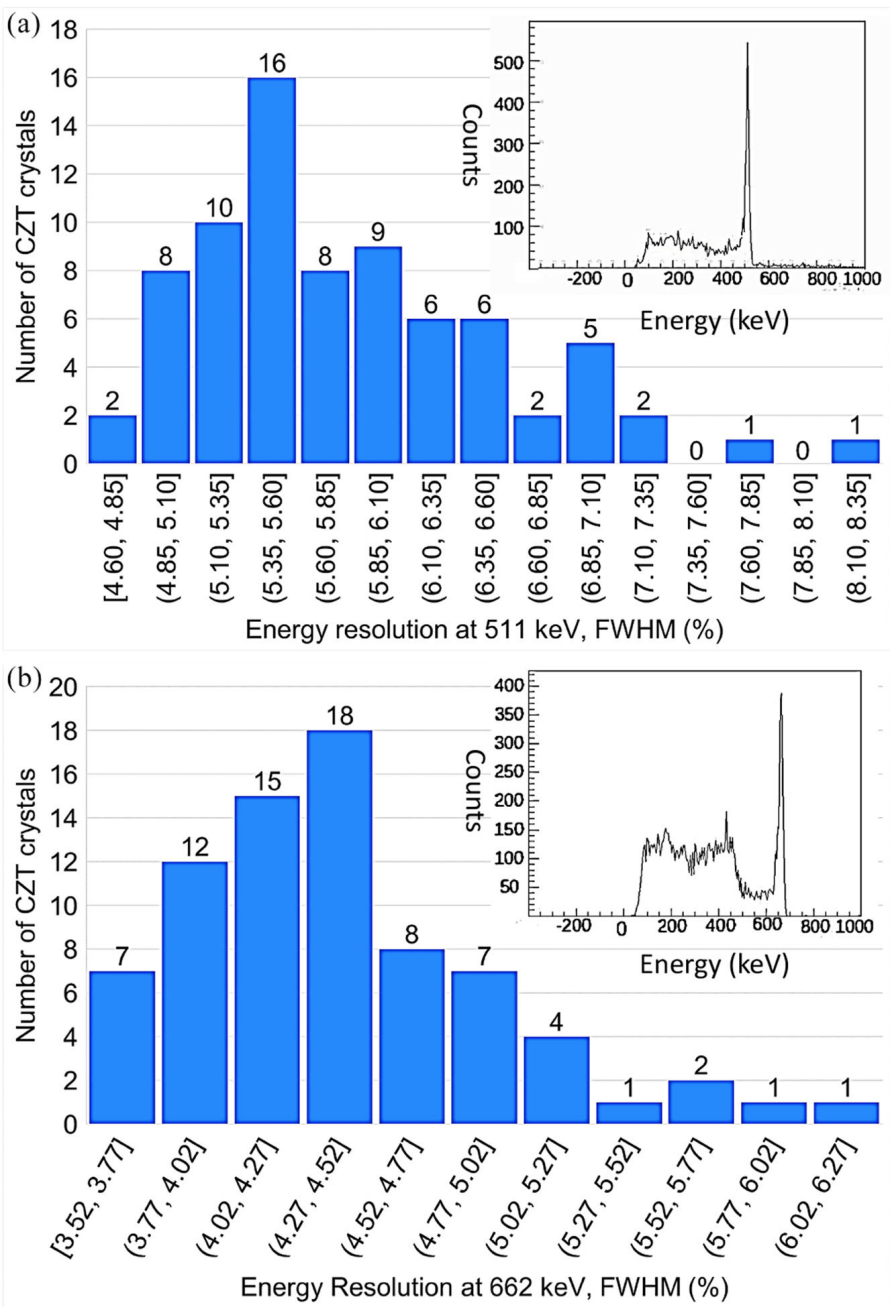
(a) Histogram of 511 keV energy resolution for 80 CZT crystals, Insert: Example energy spectrum for 511 keV. (b) Histogram of 662 keV energy resolution for 80 CZT crystals, Insert: Example energy spectrum for 662 keV.

**Fig. 13.**
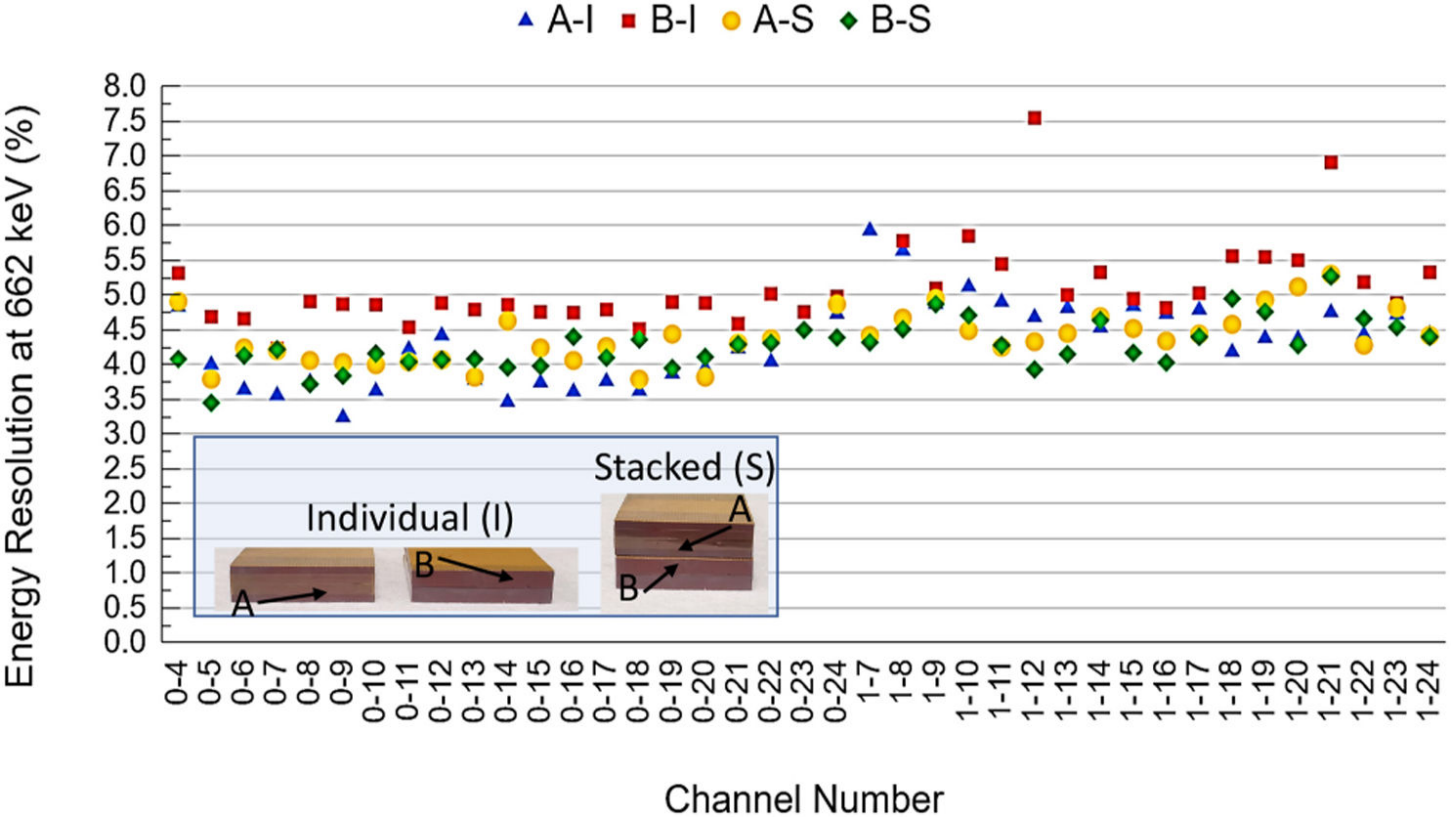
Energy resolution at 662 keV for crystals A and B, individual (A-I, B-I) and stacked (A-S, B-S) Inset: Individual and stacked testing configurations.

The energy resolution of the scaled-up system was measured after populating 80 CZT crystals in a single panel housing (Fig. 11). The average energy resolution was 4.40% ± 0.53% at 662 keV and 5.85% ± 0.73% at 511 keV. The distribution of the CZT crystal energy resolutions for both 662 and 511 keV is shown in Fig. 12. Examples of the measured energy spectrum for both energies are shown in the insets of Fig. 12. To confirm that crosstalk between modules does not have a large effect on the energy resolution, the energy resolutions of the channels on two different crystals (A and B) were measured individually and in a stack with crystal A on top of crystal B with anode flexible circuits between them (Fig. 13). The average energy resolution over 39 channels at 662 keV for crystals A and B were 4.32% ± 0.61% and 5.11% ± 0.62%, respectively. When placed in a stack, crystals A and B had an average energy resolution of 4.39% ± 0.37% and 4.28% ± 0.35%, respectively.

## IV. Discussion

The effect of flexible circuit bonding and system integration on energy resolution was studied for cross-strip CZT detectors. The steering electrode bias and RENA ASIC shaper settings were varied to find the optimum settings for lowest FWHM energy resolution at 662 keV. The FWHM energy resolution of 110 CZT crystalss were compared pre-and post-bonding. A system scale-up was also completed with 80 CZT crystals.

### A. Parameter Optimization

By optimizing the steering electrode bias and the shaper settings in the RENA ASIC, the energy resolution can be improved. When a negative potential is applied to the steering electrodes, an electric field is created that steers the mobile electrons in the CZT crystal toward the anodes and away from the gaps between the anodes [2]. The results in Fig. 7 highlight that the best energy resolution can be obtained where the steering electrode bias voltage is in range of −40 to −80 V with a compromise of leakage current contribution of steering electrode. Based on the result shown in Fig. 7, steering voltage of −60 V has been used for the rest of the experiment.

As shown in Figs. 8 and 9, as the shaper setting is increased the number of total counts is increased. Much of this increase can be attributed to an increase in noise. To optimize the system, it is desirable to maximize the fraction of photopeak counts compared to the total counts, so the noise is reduced while increasing the photopeak events. When the shaper setting is set to 0.71 *μ*s the fraction of photopeak counts accounts for only 15% of the total counts. The maximum photopeak fraction occurs at 1.1 *μ*s where the fraction of photopeak events accounts for 17.6% of the total counts (closer to the real fraction of our crystal geometry). A shaper setting of 1.1 *μ*s has been used for the remainder of the experiment.

### B. Prebonded and Post-Bonded Energy Resolution Comparison

Prebonded crystals were measured directly on the cross-strip electrode pattern without flexible circuit bonding. The post-bonded crystals had flexible circuits bonded to the crystals and the system utilized additional electronics for readout of the energy resolution. The additional electronics have been described previously and include an intermediate board for application of the high voltage and steering voltage, an RENA board with RENA ASIC, and a Fan-in board for clock distribution [14]. These electronics have been shown to have an electronic noise level of 3.45% +/− 0.52% with the application of high voltage and steering voltage [14]. This electronic noise limits the minimum energy resolution that can be measured using this system. With the combination of both the flexible circuit bonding and the additional electronics, the overall effect on the energy resolution was shown to be an average increase of 1.26% at 662 keV. There are a few cases, such as number 69 or 73, wherein the post-bonded CZT crystals have an equal or better energy resolution than the prebonded CZT crystals. Among 110 CZT crystals tested, only very few were observed to have this behavior, but we believe this is due to measurement error.

### C. Scaled-Up Energy Resolution

The scaled-up system has slightly better average energy resolution compared to the average of individually tested crystals utilizing Fig. 6(b). This is due to better grounding and shielding of the detectors when they were in the panel shown in Fig. 11. The average energy resolution for the scaled-up system was 5.85% and 4.40% at 511 and 662 keV, respectively. The difference between the individually measured crystals and the scale-up system is 0.36% for 662 keV and −0.03% for 511 keV. This shows there is no significant differences working individually or in stacked configuration.

The channel-to-channel energy resolution for individual and stacked measurements for both crystal A and B are shown in Fig. 13. There is very little change in the energy resolution of the anode channels when tested individually and in a stack. Crystal A had a 0.07% increase in energy resolution at 662 keV when tested in a stack, while the energy resolution of Crystal B decreased by 0.83% when tested in a stack. These results suggest there is not crosstalk between the anode channels of the CZT modules when stacked.

## V. Conclusion

Energy resolution is an important benefit of utilizing CZT detectors in PET systems. We have measured the energy resolution of 110 CZT crystals individually before and after bonding to flexible circuit. It was demonstrated that the flexible circuit bonding and large-scale readout electronics increases the average FWHM energy resolution at 662 keV from 3.5% ± 0.59% (tested individually) to 4.4% ± 0.53% (in an scale-up system) due to additional capacitance and cross-talk effects. Optimization of steering electrode bias and shaper settings resulted in an improvement in energy resolution and a reduction of noise compared to our previous work. Our work indicates that system integration of cross-strip CZT crystals into a stacked panel does not have a detrimental effect on the energy resolution of the CZT detector modules.
